# The Design of Large Scale IP Address and Port Scanning Tool

**DOI:** 10.3390/s20164423

**Published:** 2020-08-08

**Authors:** Chao Yuan, Jinze Du, Min Yue, Tao Ma

**Affiliations:** 1 Institute of Modern Physics, Chinese Academy of Sciences, Lanzhou 730000, China; yc2015@impcas.ac.cn (C.Y.); yuemin@impcas.ac.cn (M.Y.); matao@impcas.ac.cn (T.M.); 2 School of Computer and Communication, Lanzhou University of Technology, Lanzhou 730000, China

**Keywords:** port scanning, HIRFL, Golang, GTK, TCP

## Abstract

The control network is an important supporting environment for the control system of the heavy ion accelerator in Lanzhou (HIRFL). It is of great importance to maintain the accelerator system’s network security for the stable operation of the accelerator. With the rapid expansion of the network scale and the increasing complexity of accelerator system equipment, the security situation of the control network is becoming increasingly severe. Port scanning detection can effectively reduce the losses caused by viruses and Trojan horses. This article uses Go Concurrency Patterns, combined with transmission control protocol (TCP) full connection scanning and GIMP Toolkit (GTK) graphic display technology, to develop a tool called HIRFL Scanner. It can scan IP addresses in any range with any ports. This is a very fast, installation-free, cross-platform IP address and port scanning tool. Finally, a series of experiments show that the tool developed in this paper is much faster than the same type of software, and meets the expected development needs.

## 1. Introduction

With the rapid development of computer technology, information networks have become an important guarantee for social development. The Internet has become an indispensable tool for life. Economic, cultural, social activities, and military development are strongly dependent on the Internet. With the development of the fourth industrial revolution, network security issues have become increasingly prominent, which not only seriously hinder the development of social informatization, but also further affect the security and economic growth of the entire country. The security and reliability of the network system have become a focus of the world.

Common network attacks can be divided into four types: fake message attacks, exploitable attacks, denial of service attacks, and information gathering attacks [1]. Among them, the information collection does not cause harm to the target itself, and such attacks are used to provide useful information for further intrusions. Information collection technology is a double-edged sword. On one hand, an attacker needs to collect information before an attack to carry out an effective attack. On the other hand, a network administrator can use information collection technology to discover system vulnerabilities and repair them in advance [2,3,4]. Network administrators usually do not hide their identities during scanning. On the contrary, attackers hide their identities. The most common information collection technology is scanning technology [5,6], which includes architecture detection and utilization of information services.

There are 65,536 ports provided by TCP/IP protocol for an IP address in the computer [7]. Among them, the range of Well Known Ports is from 0 to 1023, the range of Registered Ports is from 1024 to 49,151, and the range of dynamic ports is from 49,152 to 65,535. Based on the port scanning technology, a large number of scanners have been developed. For example, OS/MVT developed by IBM [8], the 1100 series developed by UNIVAC [9], the SATAN commercial port scanner developed by Dan Farmer and Weitse Ven [10], and the Nessus system developed by Tenable Network Security [11]. Angry IP scanner and Scanrand are very fast IP address and port scanners, which were written by Anton Keks and Dan, respectively. Scanrand reduces reliability in exchange for faster scanning speed. Other port scanning tools include unicornscan, knocker, fast port scanner, etc. At present, the most widely used open-source scanner is Nmap [12,13,14], which provides a variety of scanning methods that can group multiple target IP addresses for scanning, but the disadvantage is that the scan results of the host can only be provided after the scan of the entire group is completed. In addition, at the Usenix International Security Symposium held on 27 March 2015, Durumeric and others from the University of Michigan in the United States proposed a scanner Zmap using stateless scanning technology. It can scan all IPv4 addresses in 45 min, which is 1300 times faster than Nmap, but its disadvantage is that it cannot find all the vulnerabilities, only one port can be scanned at a time, and it cannot cover devices using IPv6 protocol [15,16]. Masscan, which can scan the entire network in 6 min, also has a high usage rate and is currently the fastest port scanner [17,18]. Zmap and Masscan can be run under Linux and Mac OS, but Cygwin, WinPcap, and other tools are required when using with Windows, which brings difficulties to ordinary users.

Each scanner has its own advantages, but also has certain defects. Therefore, this paper combines the traditional scanning technology with the high concurrency of the Golang language to design a comprehensive cross-platform scanning system which can obtain more information about network security and provide better information support.

This paper is organized as follows. Section 2 describes the Common Technology of HIRFL Scanner, Section 3 introduces the overall architecture of the software, and the experimental results with detailed discussion are displayed in Section 4. Finally, Section 5 concludes this paper with a discussion on the contribution of this paper.

## 2. The Common Technology of HIRFL Scanner

### 2.1. High Concurrency of Golang Programming Language

Golang is an open-source programming language that makes it easy to build simple, reliable, and efficient software. It is a statically strongly typed, compiled language developed by Robert Griesemer, Rob Pike, and Ken Thompson of Google. It can check the most hidden program problems during compilation. Golang can be directly compiled into machine code without relying on other libraries. It uses a multi-thread model. In more detail, it is a two-level thread model. The main reason why we chose Golang to develop HIRFL Scanner is its high concurrency. Concurrency means that two or more tasks are executed within a period of time. We do not care whether these tasks are executed at a certain point in time; these tasks may or may not be executed at the same time. We only care about whether two or more tasks are solved in a short period of time (one second or two seconds). Parallel (parallelism) means that two or more tasks are executed at the same time. Concurrency is a logical concept, while parallel emphasizes the physical running state, so concurrency includes parallelism.

Go implements two forms of concurrency [19,20]. The first is multi-threaded shared memory, similar to programming languages such as Java or C++. The other is the communicating sequential processes (CSP) concurrency model. This article uses the CSP model for development, which uses communication to share memory.

**Figure 1:**
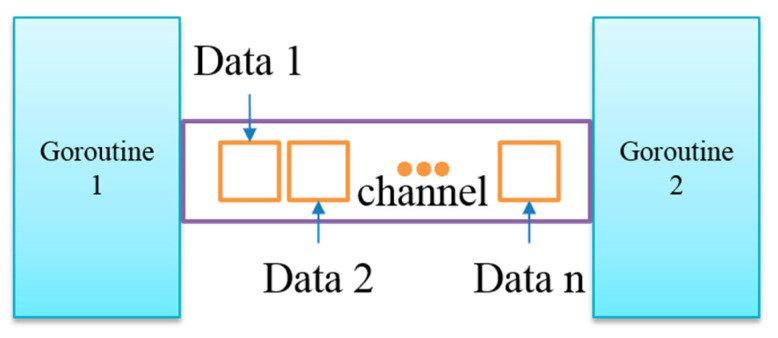
The communication process of two goroutines.

Goroutine and channel are the key components of concurrency in the CSP model. Golang encapsulates system threads (kernel-level threads) and exposes a lightweight coroutine named goroutine (user-level threads) for users. Golang’s runtime is responsible for scheduling user-level threads to kernel-level threads. The advantage of goroutine is that the context switching is performed in the complete user mode, and there is no need to switch between the user mode and the kernel mode as frequently as threads, which saves resource consumption. Golang provides a keyword “go” to create a Golang coroutine. The Go coroutine is started when we add a keyword “go” before the function or method, and thus the function or method will be Run in Go coroutines. The channel is a communication channel between various concurrent structures in the Golang language, similar to the channel in Linux. As shown in Figure 1, in the communication process of two goroutines, the buffered channel is generally used for data transmission.

### 2.2. GTK

The GIMP Toolkit (GTK) is an open-source, multi-platform-oriented GUI toolkit whose source code is distributed under the LGPL license agreement. It was originally developed by Peter Mattis and Spencer Kimball for the GNU Image Manipulation Program (GIMP) to replace the paid Motif. At present, it is one of the mainstream development tools for GUI development and has been applied to more and more programs. Unlike other GUI tools such as Qt, wxWidgets, and FLTK, GTK is completely implemented in C language.

GTK+ can be considered as the latest version of GTK. GTK contains three sets of function libraries, including libglib, libgdk, and libGTK. These libraries do not use an object-oriented mechanism, so components cannot be reused, and the message mechanism is implemented using a standard callback mechanism, while the current GTK+ uses a signal mechanism.

GTK+ is also implemented in C language; however, in terms of design, object-oriented design (OOD) is adopted flexibly. The program interface written in GTK+ is similar to Motif, which is an industry-standard GUI [21,22]. GTK+ contains many frequently-used widgets, such as file selection, color selection components, and so on. In addition, GTK+ provides some unique components, such as buttons with sub-component instead of labels, and almost any widget can be placed on such buttons. GTK+ allows software developers to show what they want in a simple way. GTK+ provides a good processing tool for the internationalization (i18n) and localization (i10n) of the application, which allows the program to be edited without modification, and only needs to switch the language data files required by different languages. Therefore, it can be used by people of different languages.

As the developer of GTK+, the GNU organization allows anyone to use all its features for free. GTK+ is portable and has multiple language front ends, such as C++, Perl, Python, TOM, Ada95, Free Pascal, Eiffel, JAVA, and C#, etc. In this article, we use GTK+3.6 to develop the display interface of the HIRFL Scanner.

### 2.3. ICMP Protocol

ICMP is the abbreviation of the Internet Control Message Protocol. It is a sub-protocol of the TCP/IP protocol suite and is used to transfer control messages between IP hosts and routers, including reporting errors, exchanging restricted control, status information, and so on. The ICMP protocol is a connection-free network layer protocol, which is extremely important for network security. When the IP data cannot access the target or the IP router cannot forward the data packet at the current transmission rate, it will automatically send the ICMP message. When we want to evaluate the network connection status, ICMP is a very useful protocol.

The ping program uses the ICMP protocol to detect whether the hosts can communicate with each other. If the ping cannot reach a host, it indicates that it cannot establish a connection with this host. It sends an ICMP echo request message to the destination host. The destination host must return an ICMP echo response message to the source host. If the source host receives a response within a certain time, the destination host is considered reachable. It works as follows:

(1) The ping command will build a fixed format ICMP request packet, and then the ICMP protocol will hand this packet to the IP layer protocol along with the destination host’s IP address. Ping can calculate the RTT (round trip time), which inserts the sending time in the data part of the packets.
(2) The IP layer protocol takes the local IP address as the source address, appending some other control information, and constructs an IP packet. After finding the MAC address corresponding to the destination IP address in a mapping table, the packet will be handed over to the data link layer. If the destination host and the source host are not in the same network segment, this will turn to the routing process.
(3) Construct a data frame at the data link layer, along with some control information. The destination address is the MAC address passed from the IP layer, and the source address is the physical address of the machine. Then, transfer them out according to the media access rules of Ethernet.
(4) After receiving the data frame, the destination host first checks its destination address and compares it with the physical address of the machine. If it matches, the data frame will be received; otherwise, the data frame will be discarded. After receiving, the destination host will check the data frame, extract the IP data packet from the frame, and give it to the local IP layer protocol. Similarly, after checking at the IP layer, the useful information is extracted and handed over to the ICMP protocol. After the latter process, the ICMP response packet is immediately constructed and sent to the source host.

### 2.4. TCP Full Connection Port Scanning Technology and Classification

Port scanning scans a section of the target host’s port or any designated ports one by one to determine which ports of the target host are open [23,24,25,26,27]. Through the open port, we can find possible vulnerabilities in the target host and fix them in time. Therefore, the scan of the host port can help us better understand the target host and is the first step to doing a good job of strengthening security.

**Figure 2:**
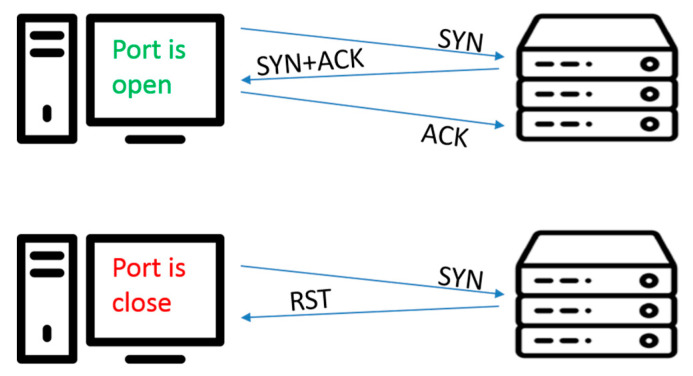
TCP three-way handshake port scanning.

In this paper, TCP full connection technology is adopted to achieve port scanning [28,29]. The scanning host attempts (using TCP three-way handshake) to establish a regular connection with the designated port of the destination host, as shown in the following Figure 2.

(1). When establishing a connection, the client sends a syn packet (syn = j) to the server and enters the SYN_SEND state, waiting for the server to confirm. When the server receives the syn packet, it must confirm the client’s ACK (ack = j + 1), and also send a SYN packet (syn = k), that is, the SYN+ACK packet. After this process, the server enters the SYN_RECV state. If the port is closed, the RST packet will be returned.
(2). The client receives the SYN+ACK packet from the server and sends an acknowledgment packet ACK (ack = k + 1) to the server. After the packet is sent, the client and server enter the ESTABLISHED state to complete the connection establishment.

We use the dial method in the standard library of the net package to connect. The connection is started by the system call connection. For each listening port, the correct connection is returned if the port is open, otherwise a connection error is returned, indicating that the port is not accessible. In order to further improve the scanning rate, this article uses the high concurrency feature of GO to program. When using the Dial function to establish a network connection, the DialTimeout function provided by the net package will actively pass additional timeout parameters to establish a connection. In HIRFL Scanner, we set the timeout of TCP connection to 100 ms.

**Figure 3:**
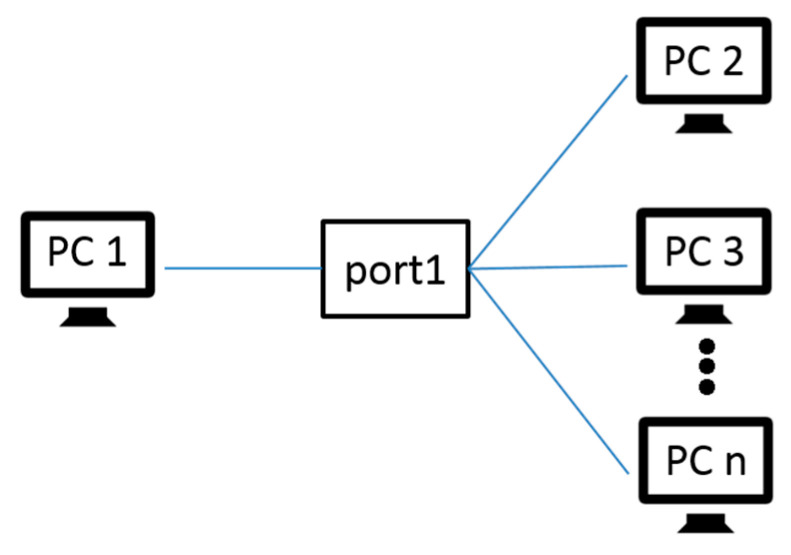
The diagram of the horizontal scanning schematic.

**Figure 4:**
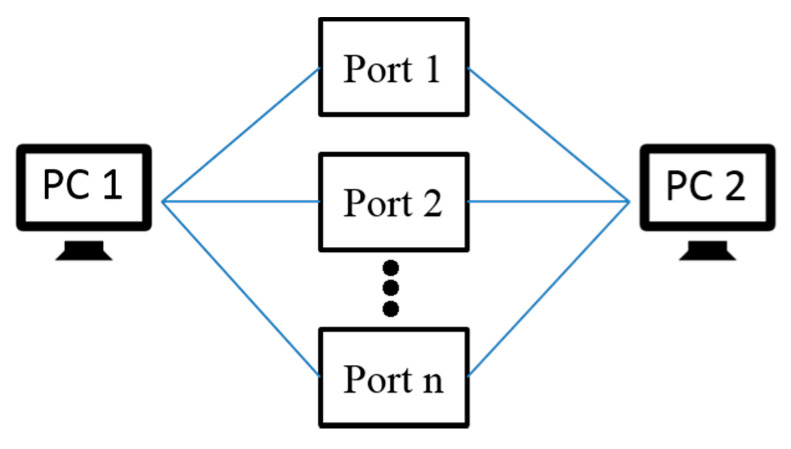
The diagram of the vertical scanning schematic.

**Figure 5:**
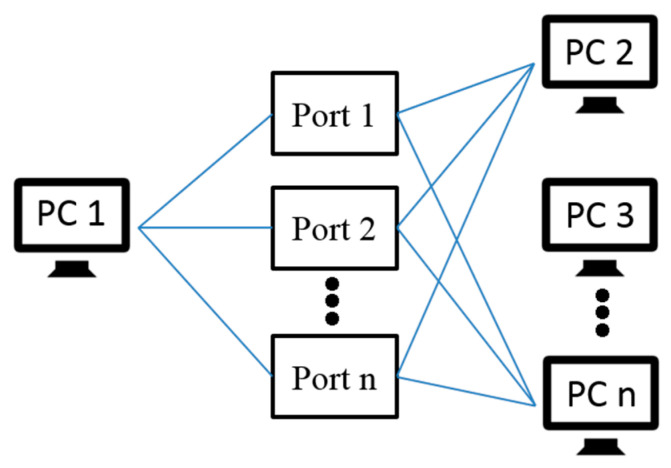
The diagram block scanning schematic.

According to different classification standards, the port scanning technology can have different classifications, such as classification according to protocol type and classification by port allocation [30,31,32]. This paper classifies the port scanning technology according to the scanning method:

(1). Horizontal scanning: For a specific port, scan different target hosts, as shown in Figure 3 below.
(2). Vertical scanning: Scan different ports for a specific host as shown in Figure 4 below.
(3). Block scanning: Block scanning is a combination of horizontal and vertical scanning. It scans multiple times for different ports of different hosts, as shown in Figure 5 below.

## 3. Structure of the HIRFL Scanner

**Figure 6:**
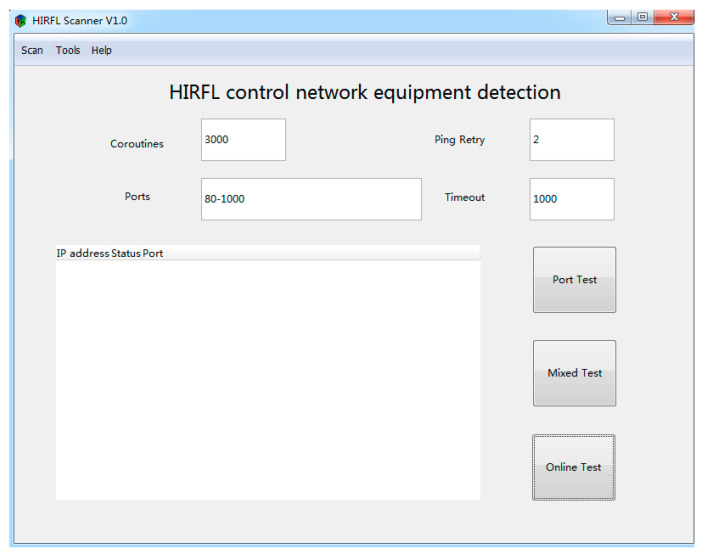
The HIRFL Scanner software’s main interface.

HIRFL Scanner is implemented in CS architecture, which is conducive to guarantee the safety and response speed of the system. The main interface of the system is shown in Figure 6 below, which is developed using GTK+3.6. It can be divided into three sub-modules: the parameter input module, function selection module, and result output module.

The parameter input module mainly enables users to input various parameters used in port scanning according to their needs. For example, regarding the number of coroutines, each goroutine occupies 2 KB of memory by default. On 32-bit processors, the maximum number of Go programs is about 80,000, but on 64-bit processors, the Go program has no limit on the number of coroutines created. In this way, the user can reasonably enter the number of coroutines based on the number of scan tasks.

The second parameter is the number of times the program repeats the ping process when the first ping scan fails. The default value of the program is 2 times. This value will also affect the scan time. Num of Port is the port number to be scanned. The program will automatically calculate the required number of TCP connections. In order to increase the speed of large-scale IP address and port scanning, the timeout period of TCP connections is 100 ms by default in this system. The function selection module is the core of this system, and it mainly includes IP address online scanning, port scanning, and mixed scanning (ip + port scanning). The user can complete the task of scanning by selecting different functions. When the system is scanning, the scanned results will be displayed in real-time in the result output module. After completing the scanning task, the system will inform the user of the final result of the scan in the form of a dialog box.

The main program is developed with go1.13.4, and the core packages are net, sync, icmp, and ipv4. Package net provides a portable interface for network I/O, including TCP/IP, UDP, domain name resolution, and Unix domain sockets. We use the DialTimeout method in the net package to receive the protocol, IP address, port number, and the timeout period. Package sync provides basic synchronization primitives such as mutual exclusion locks. Mutex is used to solve the problem of data competition, while WaitGroup solves the problem of coroutine synchronization. Package icmp provides basic functions for the manipulation of messages used in the Internet Control Message Protocols, ICMPv4 and ICMPv6. The ipv4 package is used to implement the IP level socket option for the Internet Protocol version 4. Other packages used in the development of HIRFL Scanner include bufio, os, errors, fmt, time, etc.

**Figure 7:**
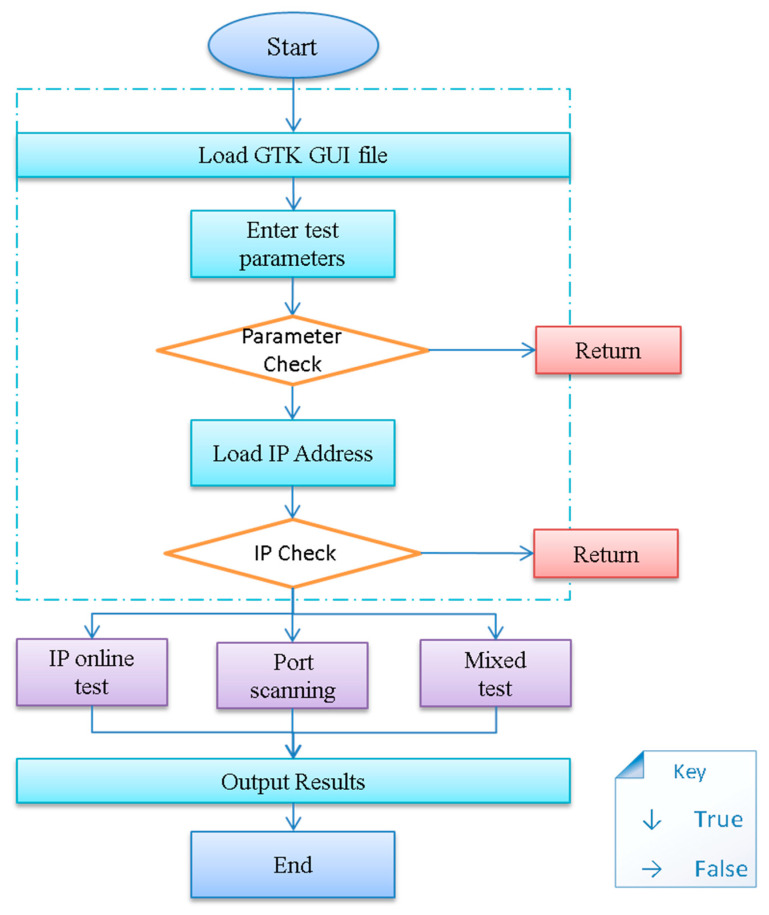
The flow chart of HIRFL Scanner.

Figure 7 shows the workflow of the system. Due to the separate design of the front and back end, the system first loads the GTK GUI graphic display file. In the process of parameter and IP address verification, a return represents that the user needs to check the input parameters or IP address. The IP address of this program is read from the TXT file.

## 4. Experiment and Result

### 4.1. Data Description and Preprocess

In order to verify the scanning rate and correctness of the HIRFL Scanner system, we conducted a series of experiments on the Lanzhou heavy ion accelerator control network and compared it with the industry-renowned scanning software Nmap and Masscan scanners. As shown in Table 1, the IP addresses to be scanned come from the HIRFL control network. There is a total of 13,915 IP addresses in 55 VLANs, excluding network addresses, broadcast addresses, and gateways. The IP address is exported from the MYSQL database to a TXT file for the scanner to load. The operating system of the HIRFL Scanner and Nmap is windows 7 64-bit, and the CPU is Intel Core I7-6567U 3.3 GHz, with 16GB memory. Masscan uses the same hardware environment, and the operating system is Centos 7. In order to improve the accuracy of the test results, all experimental results are the average values of the three tests, which were conducted under different network load periods. We use Nmap with a graphical interface Zenmap 7.80, and the version of Masscan is 1.0.6.

### 4.2. The Comparisons of IP Address Online and Port State Independent Detection

We first divide all the IP addresses on the accelerator into 6 groups by number for the ping scan test. In this experiment, Nmap was scanned in three time modes: T3 (normal), T4 (aggressive), and T5 (insane); the sP parameter is used to perform a ping scan without further testing, such as for port scans or operating system scans. Then we conducted port scanning 6 times for a server with IP address: 10.10.100.125. The number of scanning coroutines of HIRFL Scanner is 3000. The number of ping repetitions is 3, and the timeout period is 100 ms. The IP scanning results are shown in Table 2. HS Time represents the scanning time used by the HIRFL Scanner. As the number of scanning ports increases, the scanning time of the HIRFL scanner and Nmap is increasing. For Nmap, we can see that T5 mode can take the least amount of time to complete the scan. The HIRFL Scanner can complete the scanning of all IP addresses in about 44 s, and the scanning speed is significantly faster than Nmap, which finished the scanning process after 378.5 s. When the number of IPs is 2530, the HIRFL scanner is 93.19% faster than Nmap using T5 mode. Judging from the scanning results, the scanning results of the HIRFL scanner and Nmap are basically the same, and the maximum deviation is 4. This maximum deviation refers to the number of inconsistencies between the HIRFL scanner and Nmap scanning results, mainly the number of false positives.

In the vertical scanning experiment, Nmap was scanned in sS (TCP SYN) and sT (TCP connect) modes, respectively, and the time template was T5. For HIRFL Scanner, the coroutine is set to 3000 and the timeout is 100 ms. Table 3 shows the vertical scanning results of the HIRFL Scanner and Nmap Port Scanner. The data shows that the accuracy of the two port scanners is basically the same, and the deviation may be caused by packet loss. For Nmap, sS mode is significantly faster than sT. The scanning speed of the HIRFL Scanner is also better than that of Nmap. In the small-scale port scanning, the maximum speedup ratio is 98.33%, while in the full port scanning, the speed is increased by 44.68%.

### 4.3. The Comparisons of Scanning Results of IP Devices with Different Port Numbers in Accelerator Control Network

We scanned each port of all devices in the accelerator experiment. A total of 912 million ports of 13,915 devices were scanned. For Nmap, we choose T5 and sS parameters to accelerate scanning. The coroutines and timeout of the HIRFL scanner are set to 3000 and 50 ms, respectively. Table 4 summarizes the statistical results of the top ten services running on each port in this experiment. When using Nmap to scan, it took a week to complete all port scans, while the HIRFL Scanner shortened the time to 38.65 h. It can be seen from Table 4 that there are many services of HIRFL system equipment running on non-standard ports, and Nmap only scans ports from 1 to 1024 by default, and those services running on non-standard ports cannot be accurately identified. Similarly, it can be observed that the port scanning statistics of HIRFL Scanner and Nmap have deviations. The maximum deviation is 7, which may be caused by the scanning time period. The error between them is mainly based on false positives.

### 4.4. The Comparison of Hit Rate when Using the Shodan Dataset

In this experiment, we use the scanning results of Shodan [33,34] as the standard to scan devices in the Shodan database that provide FTP, SSH, Telnet, SMTP, HTTP, and POP3 services in China. According to the data in the Shodan database on 5 June 2020, there are 1,037,806 devices providing FTP services in China. We chose 10,000 of them to perform the scanning experiment, so the denominator is 10,000, and other protocols also use this configuration. The hit rate is used to evaluate the accuracy of the scanner, its definition is as follows:

(1) $$Hit\;rate=\frac{total\;number\;detected\;by\;the\;scanner}{10000}$$

**Figure 8:**
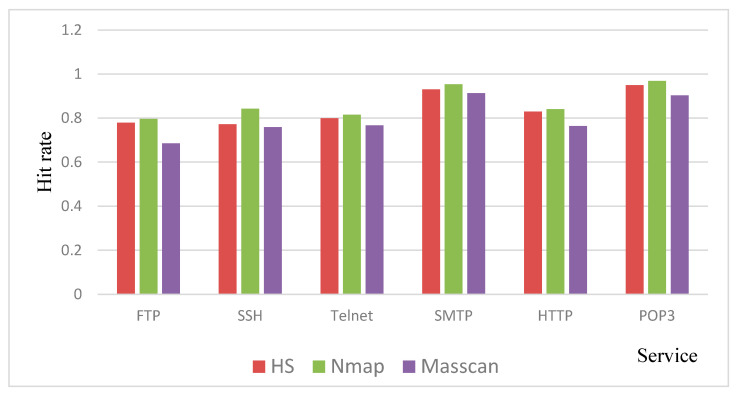
The comparison of hit rate.

Because the scanning process is performed via the Internet, there may be situations such as network congestion that affect the scanning results, so we continue to adopt the method of taking the average of three tests. The hit rate of each scanner is shown in Figure 8. For Nmap, we continue to select T5 and sS parameters to speed up the scanning. The coroutines and timeout of HIRFL Scanner are set to 3000 and 100 ms, respectively. Masscan’s packet sending speed is set to 1000 packets per second, and it has the best scanning speed performance, but the scanning accuracy is quite low. The scanning accuracy of HIRFL Scanner is basically consistent with Nmap, and the maximum error of the hit rate is 0.07. The inconsistency may be caused by network packet losses.

## 5. Conclusions

Port scanning is very useful for defensive penetration testing of HIRFL devices. Scanning HIRFL devices can determine which services are exposed to the network, therefore we can check the configuration of each device in a targeted way. In addition, we can take preventive measures to reduce the losses caused by malicious attacks. Based on the high concurrency characteristics of the Golang language, this paper develops a large-scale IP address and port scanning tool: HIRFL Scanner. The scanner adopts CS architecture and employs GTK to develop the front-end GUI interface, so as to achieve the purpose of separating the front end and back end. The most important feature of this tool is the cross-platform and user-friendly operation interface. It allows the user to specify an IP range or port number (comma separated list), and the number of goroutines the user wants to create at runtime. We used the HIRFL control network and Shodan data sets to verify the accuracy and scanning rate of the HIRFL Scanner system. Comparative experiments show that the system’s scanning rate is significantly superior to the Nmap scanner, and the accuracy is basically the same as Nmap, which meets our application needs.

## Figures and Tables

**Table 1:** All IP addresses used in port scanning.

VLAN	IP Address	VLAN_DESC	VLAN	IP Address	VLAN_DESC
10	10.10.2.0/24	Central room	107	10.10.107.0/24	CSRm
14	10.10.14.0/24	Single particle	108	10.10.108.0/24	CSRm
15	10.10.15.0/24	Single particle	109	10.10.109.0/24	CSRm
16	10.10.16.0/24	SSC	110	10.10.110.0/24	CSRm
17	10.10.17.0/24	SSC	111	10.10.111.0/24	CSRm
41	10.10.41.0/24	T128	112	10.10.112.0/24	CSRm
42	10.10.42.0/24	T128	113	10.10.113.0/24	Langdao North
43	10.10.43.0/24	T128	114	10.10.114.0/24	Langdao North
45	10.10.45.0/24	SFC	115	10.10.115.0/24	Langdao North
46	10.10.46.0/24	SFC	116	10.10.116.0/24	Langdao South
48	10.10.48.0/24	SFC	117	10.10.117.0/24	Langdao South
51	10.10.51.0/24	HIRFL	118	10.10.118.0/24	Langdao South
52	10.10.52.0/24	HIRFL	119	10.10.119.0/24	Langdao South
53	10.10.53.0/24	HIRFL	120	10.10.120.0/24	RIBLL2
54	10.10.54.0/24	HIRFL	121	10.10.121.0/24	RIBLL2
90	10.10.90.0/24	HIRFL	122	10.10.122.0/24	RIBLL2
91	10.10.91.0/24	HIRFL	123	10.10.123.0/24	RIBLL2
92	10.10.92.0/24	HIRFL	124	10.10.124.0/24	RIBLL2
95	10.10.95.0/24	HIRFL	125	10.10.125.0/24	RIBLL2
96	10.10.96.0/24	HIRFL	126	10.10.126.0/24	CSRe
99	10.10.99.0/24	RIBLL1	127	10.10.127.0/24	CSRe
100	10.10.100.0/24	RIBLL1	128	10.10.128.0/24	CSRe
101	10.10.101.0/24	RIBLL1	129	10.10.129.0/24	CSRe
102	10.10.102.0/24	CSRm	130	10.10.130.0/24	CSRe
103	10.10.103.0/24	CSRm	131	10.10.131.0/24	CSRe
104	10.10.104.0/24	CSRm	998	172.16.110.0/24	virtualization
105	10.10.105.0/24	CSRm	999	172.16.100.0/24	virtualization
106	10.10.106.0/24	CSRm			

**Table 2:** Scanning results of HIRFL Scanner and Nmap IP Scanner.

Num of IP	HS Time (s)	Nmap Time (s)			HS Result	Nmap Result		
		T3	T4	T5		T3	T4	T5
253	4.12	21.83	21.53	20.77	47	47	47	47
2530	5.98	102.85	96.77	87.75	201	201	201	201
5060	11.45	184.58	175.52	159.10	442	441	441	441
7590	16.57	267.90	247.58	228.15	657	656	656	656
10,120	30.42	329.27	311.39	286.92	843	839	840	841
13,915	44.08	436.21	415.03	378.50	1143	1140	1139	1140

**Table 3:** The vertical scanning results of HIRFL scanner and Nmap.

Num of Ports	HS Time (s)	Nmap T5 Time (s)		HS Result	Nmap T5 result	
		sS	sT		sS	sT
1000	0.31	18.77	65.10	9	9	9
10,000	4.12	21.36	251.92	12	12	12
20,000	7.32	23.68	456.72	11	12	12
30,000	10.84	27.18	662.92	13	13	13
40,000	14.99	29.00	868.51	15	15	15
65,535	20.65	37.34	1391.98	16	17	17

**Table 4:** The block scanning results of HIRFL Scanner and Nmap.

Port	Service	HS Count	Nmap Count	Variance
80	http	262	256	6
3389	ms-wbt-server	155	148	7
445	microsoft-ds	143	147	4
22	ssh	139	143	4
111	rpcbind	107	110	3
49,152	unknow	106	110	4
23	telnet	102	107	5
49,153	unknow	99	101	2
5064	channel access	92	97	5
59,110	ni-psp	80	83	3
